# Supplementary material for: Cancer Stem Cells and Somatic Stem Cells as Potential New Drug Targets, Prognosis Markers, and Therapy Efficacy Predictors in Breast Cancer Treatment
Source: Biomedicines. 2021 Sep 14;9(9):1223. doi: 10.3390/biomedicines9091223 (PMC8467941; doi:10.3390/biomedicines9091223)
Supplement: Supplementary file 1 [file biomedicines-09-01223-s001.zip › biomedicines-1359980-supplementary.pdf]

Table S1. Clinical and morphological parameters of the patients included in the study.

Patients#	Age	Histological Type and Grade	Estrogen Receptor Status	Progesterone Receptor Status	HER2 Receptor Status	Lymph Node Metastasis Status	Ki67, %	MFS[1], month
Patient#1	59	Invasive carcinoma grade 2, Stage: T2N1M0	8	Negative	1+	Negative	45	6
Patient#2	54	Invasive carcinoma grade 1, Stage: T1NxM0	7	5	1+	Negative	32	12
Patient#3	39	Invasive ductal carcinoma grade 2, Stage: T2N2M0	7b	Positive	Negative	Negative	35	2
Patient#4	42	Invasive carcinoma grade 2, Stage: T2N1M0	Negative	Negative	Negative	Positive	65	20
Patient#5	63	Invasive lobar duct cancer with DCIS comedic type foci, a multicentric form of tumor growth. grade 2, Stage: T2N2M0	8	4	Negative	Positive	42	12
Patient#6	57	Invasive carcinoma, grade 2, Stage: T2N0M0	8b	4b	3+	Negative	37	5
Patient#7	49	Invasive carcinoma, grade 2, Stage: T2NxMx	Negative	Negative	Negative	Negative	38	24
Patient#8	60	Invasive carcinoma, grade Luminal B, Stage: T2N1Mx	Negative	Negative	Negative	Negative	45	12

Patient#9	38	Invasive carcinoma grade 2, Stage: T2N1M0	Negative	Negative	Negative	Negative	41	20
Patient#10	42	Invasive carcinoma grade 2, Luminal B, Stage: T2N0M0	Negative	Negative	Negative	Positive	62	10
Patient#11	66	Invasive carcinoma grade 2, Stage: T3N2M0	7	Negative	1+	Negative	35	24
Patient#12	38	Invasive carcinoma grade 2, Stage: T2N1M0	8	Positive	Negative	Negative	44	24

Abbreviations: [1]MFS, metastasis-free survival.
